# Effectiveness and safety of mycophenolate mofetil in idiopathic pulmonary fibrosis

**DOI:** 10.1371/journal.pone.0176312

**Published:** 2017-04-25

**Authors:** Anoop M. Nambiar, Antonio R. Anzueto, Jay I. Peters

**Affiliations:** 1 Department of Medicine, Division of Pulmonary Diseases and Critical Care Medicine, University of Texas Health Science Center San Antonio, San Antonio, Texas, United States of America; 2 Department of Medicine, Division of Pulmonary Diseases and Critical Care Medicine, South Texas Veterans Health Care System, San Antonio, Texas, United States of America; Universitatsklinikum Freiburg, GERMANY

## Abstract

**Background:**

Currently available antifibrotic treatments may slow down disease progression in idiopathic pulmonary fibrosis (IPF), but are associated with potentially significant side effects and are costly. Mycophenolate mofetil (MMF) is well known for its potent immunosuppressive properties and possesses important antiproliferative and antifibrotic effects. The safety and effectiveness of MMF in IPF is unknown.

**Methods:**

We performed a retrospective multicohort analysis of IPF patients treated with MMF compared to those treated with either ineffective/harmful treatments or no treatment. Longitudinal change in forced vital capacity (FVC) between the groups was analyzed using a mixed model with random intercept and slope allowing for repeated measures within subjects. Categorical change in FVC, median overall survival, and adverse events were also assessed.

**Results:**

Forty-one IPF patients were included: 11 treated with MMF, 20 treated with ineffective/harmful agents (such as prednisone, azathioprine, and/or NAC), and 10 did not receive any specific treatment for their IPF. After one year, there was a trend towards reduced FVC decline in the MMF-treated group (-76.3 mL, -2.4% of predicted) compared to the non-MMF-treated (-165 mL, -8.9% of predicted) and the no-treatment (-239 mL, -11.5% of predicted) groups, respectively. By categorical change, there was a trend towards greater FVC stability in the MMF-treated group (87.5%) compared to the non-MMF-treated (57%) and the no-treatment groups (50%), respectively. MMF-treated IPF patients had a trend towards improved median overall survival (40.3 months) compared to the non-MMF-treated (25.5 months) and the no-treatment (29.3 months) groups, respectively. Treatment-related adverse events were not different between groups; however, very few adverse events were reported overall.

**Conclusions:**

MMF treatment was associated with potentially clinically important trends toward reduced annual FVC decline (similar to approved antifibrotics), greater FVC stability and improved overall survival in IPF patients. MMF was generally safe, well tolerated, and relatively inexpensive. Future prospective studies of MMF in combination with antifibrotic therapy in IPF are needed.

## Background

Idiopathic pulmonary fibrosis (IPF) is a chronic, progressive, and inevitably fatal fibrotic lung disease of unknown cause associated with a median survival of less than five years from time of diagnosis—a prognosis worse than many common cancers [1,2]. No pharmacologic therapy has been proven to stop or reverse disease progression. Lung transplantation is the only known cure but is only an option for highly selected, younger, healthier patients and is associated with a median survival of 5 years post-transplant. For years, treatment for IPF consisted of potent combinations of corticosteroids, immunosuppressive drugs such as azathioprine or cyclophosphamide, and/or an antioxidant such as N-acteylcysteine based on consensus-based guidelines and small flawed clinical trials lacking true placebo control groups [3,4]. In late 2011, the prednisone, azathioprine, and N-acetylcysteine arm of the PANTHER-IPF trial was stopped early due to more deaths, hospitalizations, and adverse events compared to placebo [5]. This subsequently led to a moratorium on the use of corticosteroids and all immunosuppressive agents specifically for the treatment of IPF. In 2014, two antifibrotic therapies, pirfenidone and nintedanib, were shown in large, phase three, multicenter, randomized controlled trials to significantly reduce annual FVC decline (approximate absolute reduction of 100 mL and relative reduction of 50%) in patients with mild-to-moderate IPF (as defined by FVC > 50%, DLCO > 30%) [6,7]. As a result, regulatory agencies all around the world, including the U.S. Food and Drug Administration, simultaneously approved both pirfenidone and nintedanib for the treatment of IPF in October 2014. Although these drugs are generally considered safe and well tolerated, they both may only slow down IPF disease progression in some patients and are very expensive with wholesale prices of nearly $95,000 per patient for one year of treatment without insurance coverage or financial assistance [8–10]. Future novel therapies for IPF currently remain in phase 2 trials and, if successful, are only likely to become available in no less than five years [11]. As such, there clearly remains an unmet need for more effective, safer, and less expensive treatments than the currently available antifibrotics for IPF patients.

Mycophenolate mofetil (MMF, CellCept®), a prodrug of mycophenolic acid (MPA), is a potent selective non-competitive inhibitor of inosine monophosphate dehydrogenase (IMPDH), thereby interfering with purine synthesis preferentially in T and B lymphocytes [12]. Subsequent inhibition of lymphocyte proliferation leads to suppression of cell-mediated immune responses and antibody formation. As a result of its potent immunosuppressive properties, MMF is a commonly employed antimetabolite FDA-approved for the prophylaxis against rejection of multiple organs (renal, cardiac, and liver) [13] as well as off-label use for prophylaxis against lung transplantation rejection [14], treatment of chronic rejection of lung transplantation [15], retroperitoneal fibrosis [16], rheumatoid arthritis [17], granulomatosis with polyangiitis [18], sarcoidosis [19], and systemic lupus erythematosis, with or without renal involvement [20–23]. Beyond its anti-inflammatory effects and unlike other immunosuppressives such as azathioprine, there is evidence to suggest that MMF may also have important antiproliferative and antifibrotic effects [24–28]. As a result, there is growing use of MMF in various interstitial lung diseases, such as related to connective tissue diseases (e.g., rheumatoid arthritis, scleroderma) [29–38] and chronic hypersensitivity pneumonia [39], all of which may share underlying fibrotic histopathology similar to IPF. However, despite a theoretical basis for its use [40], there exists only one small published case series of ten IPF patients treated with MMF that showed no beneficial effect on physiologic or radiologic parameters at 6 and 12 months post-treatment without any significant adverse events reported [41]. Notably, this study was small, under-powered, and lacked any comparator control group to detect a potentially clinically important treatment effect. Considering MMF’s demonstrated immunomodulatory, antiproliferative, and antifibrotic effects in a variety of inflammatory and fibrotic diseases such as ILD as well as its favorable safety profile and relative cost savings, we hypothesized that MMF would be an effective and safe treatment for patients with IPF.

## Methods

### Patients

This study was approved by the Institutional Review Board of the University of Texas Health Science Center at San Antonio (HSC20140025H) and the Research and Development Committee of the South Texas Veterans Health Care System (VA Project #0004). Waiver of consent was authorized due to anonymous analyzation of data. Following our institutional review board approval, the electronic medical records from three study sites all affiliated with the University of Texas Health Science Center (University of Texas Medicine San Antonio ILD/IPF Clinic, University Health System, and South Texas Veterans Health Care System) were searched using ICD-9 codes that identified potential patients with interstitial lung disease or idiopathic pulmonary fibrosis. Study period of interest was between January 1, 2008 and December 31, 2012 since significantly fewer IPF patients were treated with immunosuppressive drugs such as azathioprine or mycophenolate following release of the interim findings from the PANTHER-IPF study in October 2011. Prescription drug databases at each site were also reviewed to identify patients with the relevant ICD-9 diagnoses and treated with MMF during the study period. Three-hundred and sixteen patients were identified. Of these patients, only those that met 2011 ATS/ERS/ALAT/JRS IPF diagnostic criteria were included. PFT data must have been available at time zero (start of treatment) or either at six and/or twelve months. Depending on their treatment regimen, patients were then divided into three groups: 1) MMF-treated group: those treated with at least one gram per day for at least six months, 2) non-MMF-treated group: those treated with proven ineffective or harmful therapy such as corticosteroids, azathioprine, N-acetylcysteine, either as monotherapy or in some combination, and 3) no-treatment group: those patients who not treated with any specific therapy for their IPF.

### Study design and assessment

A retrospective multiple cohort analyses was performed. Data abstracted included age, gender, ethnicity, smoking history and amount, time from symptoms to diagnosis, HRCT pattern, surgical lung biopsy histopathology pattern, comorbidities, use of supplemental oxygen, evidence of recent disease worsening, presence of any autoantibodies, prior treatments, and concurrent treatments (such as corticosteroids, NAC, etc.). Available pulmonary function (PFT) data (including FVC, FEV1, TLC, DLCO) and six-minute walk data were recorded at time zero, six, and twelve months. Total duration and dosages of concurrent prednisone, if used, were recorded. Lastly, treatment-related side effects and adverse events, including drug intolerance (due to gastrointestinal complaints or other), liver function abnormalities, leukopenia, anemia, other hematologic abnormalities, infectious complications, as well as the frequency of drug intolerance, acute exacerbations (AE-IPF), hospitalizations, and death, were noted following careful chart review. AE-IPF are defined as worsening dyspnea within the past 30 days, increase in supplemental oxygen requirements, new bilateral infiltrates on chest imaging, and the absence of an identifiable and potentially more treatable cause (e.g. infection/pneumonia, pulmonary embolism, pneumothorax, congestive heart failure) [42]. Vital status will be assessed via review of the electronic medical record, internet obituary search, and query of the Social Security Death Index.

### Statistical analysis

Univariate descriptive statistics were used to analyze patient characteristics. Continuous data are presented as either means with standard deviations or medians with inter-quartile ranges. Group differences were assessed using Fisher’s Exact Test for categorical variables and the Kruskal-Wallis test for continuous variables. A mixed model with random intercept and slope allowing for repeated measures within subjects was used to assess the change in FVC, both absolute and percentage of the predicted value, across time. For time-to-event analyses, median progression-free survival and median overall survival were estimated from Kaplan-Meier curves and treatment groups were statistically compared with the log-rank test. All reported p values are two-sided and were considered statistically significant when less than 0.05. All analyses were done using SAS 9.4 (SAS Institute, 100 SAS Campus Drive, Cary, NC, USA).

## Results

### Study patients

Out of 316 medical charts electronically reviewed, a total of 41 patients met pre-specified inclusion/exclusion criteria for this study. A major reduction in potentially eligible patients was due to inaccurate ICD-9 diagnostic codes that were used initially to identify potential patients. Eleven patients were in the MMF-treated group, 20 patients in the non-MMF-treated group (treatment with ineffective or harmful agents, such as prednisone, azathioprine, and/or NAC), and 10 patients in the no-treatment group, who did not receive any specific treatment for their IPF. Demographic and baseline characteristics are summarized in Table 1. We did not collect data on patients enrolled in either pulmonary rehabilitation or palliative care. The majority of patients in the cohorts were older (mean age 66.1+/-8.9 years), male (63.4%), white non-Hispanic (58.5%), and former smokers (78%). Although there were no statistically significant imbalances in baseline characteristics, there were less males in the MMF-treated group (6, 54.5%) compared to the non-MMF-treated (12, 60%) or the no-treatment (8, 80%) groups, respectively. The MMF-treated group had worse baseline FVC (mean 57.8+/-19.9%) compared to the non-MMF-treated (mean 63.8%+/13.8%) and the no-treatment (mean 68.3+/-20.4%) groups, respectively.

**Table 1:** Baseline patient characteristics.

Characteristic	No Rx (n = 10)	Non-MMF-Rx (n = 20)	MMF-Rx (n = 11)	Total (n = 41)
**Age—yr**	67.4 +/- 8.7	67 +/- 9.6	63.3 +/- 7.7	66.1 +/- 8.9
**Male sex–no. (%)**	8 (80)	12 (60.0)	6 (54.5)	26 (63.4)
**Ethnicity**				
**White non-hispanic–no. (%)**	7 (70)	12 (60.0)	5 (45.5)	24 (58.5)
**Hispanic–no. (%)**	2 (20)	8 (40.0)	6 (54.5)	16 (39)
**Former smoker–no. (%)**	9 (90)	17 (85.0)	6 (54.5)	32 (78)
**Time since diagnosis—mos**	18 +/- 10.2	13.7 +/- 8.9	16.7 +/- 9.9	15.5 +/- 9.5
**Lung physiological features**				
**FVC—% of predicted value**	68.3 +/- 20.4	63.8 +/- 13.8	57.8 +/- 19.9	63.3 +/- 17.3
**DLCO—% of predicted value**	45.2 +/- 17.4	42.7 +/- 19.7 (of 18 pts)	44.1 +/- 15.7 (of 10 pts)	44.1 +/- 17.5 (of 39 pts)
**Use of supplemental oxygen–no. (%)**	8 (80)	19 (95)	8 (72.7)	35 (85.4)
**High-resolution CT findings–no. (%)**				
**Definite UIP pattern**	9 (90)	18 (90.0)	10 (90.9)	37 (90.2)
**Possible UIP pattern**	1 (10)	2 (10.0)	1 (9.1)	4 (9.8)
**Surgical lung biopsy–no. (%)**	5 (50)	6 (30)	1 (9.1)	12 (29.3)

Plus-minus values are means +/- standard deviations. There were no statistically significant differences between the three groups in any of the baseline characteristics shown. FVC denotes forced vital capacity, FEV1 forced expiratory volume in one second, TLC total lung capacity, DLCO diffusion capacity of carbon monoxide, UIP usual interstitial pneumonia.

Various treatment combinations were employed in both the MMF-treated and non-MMF-treated groups as displayed in Table 2. In the MMF-treated group, a combination of MMF, prednisone, and NAC was the most frequent regimen (6, 54.5%). This was followed by MMF and NAC in 3 patients and MMF monotherapy in 2 patients. In the non-MMF-treated group, NAC alone was the most frequent regimen (9, 45%) followed by prednisone, azathioprine, and NAC (4, 20%) and prednisone alone (4, 20%). Average prednisone dose among those treated with it was lower, but not statistically different, between the MMF-treated (10 +/- 2.7 mg/day) compared to the non-MMF-treated groups (13.3 +/- 5.6 mg/day). Reported comorbidities and their frequency amongst all subjects are displayed in Table 3. There were no significant differences between groups. In addition, low-level ANA positivity and low-level rheumatoid factor was noted in only 2 patients in the MMF-treated, 1 in the non-MMF-treated, and 0 in the no-treatment groups, respectively. Per the 2011 ATS/ERS/ALAT/JRS IPF guidelines, all patients with significant clinical and/or serologic evidence of a connective tissue disease were excluded.

**Table 2:** Treatment combinations of the MMF-treated and non-MMF-treated groups.

Treatment	Non-MMF-Rx (n = 20)–no. (%)	MMF-Rx (n = 11)–no. (%)
**MMF, prednisone, NAC**		6 (54.5)
**MMF and NAC**		3 (27.3)
**MMF only**		2 (18.2)
**NAC only**	9 (45)	
**Prednisone, azathioprine, NAC**	4 (20)	
**Prednisone only**	4 (20)	
**Prednisone and azathioprine**	1 (5)	
**Prednisone and NAC**	1 (5)	
**Azathioprine and NAC**	1 (5)	

**Table 3:** Comorbidities (ordered by frequency).

	No Rx (n = 10)	Non-MMF-Rx (n = 20)	MMF-Rx (n = 11)	Total (n = 41)
**GERD**	8 (80)	15 (75)	10 (90.9)	33 (80.5)
**DM**	3 (30)	7 (35)	7 (63.6)	17 (41.5)
**CAD**	3 (30)	5 (25)	4 (36.4)	12 (29.3)
**PAH**	2 (20)	4 (20)	2 (18.2)	8 (19.5)
**OSA**	0 (0)	5 (25)	1 (9.1)	6 (14.6)
**VTE**	1 (10)	1 (5)	2 (18.2)	4 (9.8)
**COPD**	2 (20)	1 (5)	0 (0)	3 (7.3)

GERD denotes gastroesophageal reflux disease, DM diabetes mellitus, CAD coronary artery disease, PAH pulmonary arterial hypertension, OSA obstructive sleep apnea, VTE venous thromboembolism, and COPD chronic obstructive pulmonary disease.

### Primary outcome

**Fig 1:**
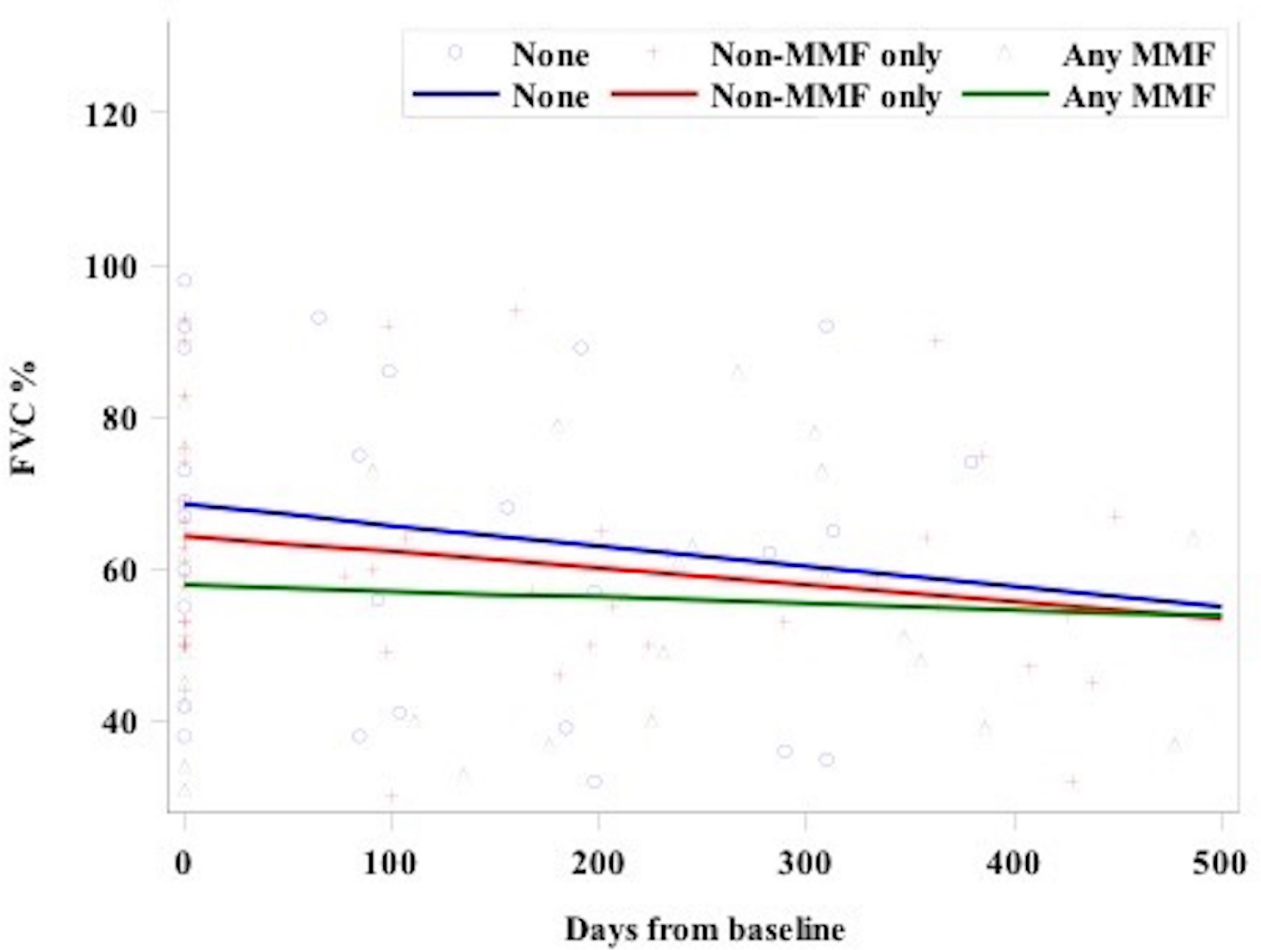
Change in FVC from baseline to 12 months. p-value for days by treatment group interaction = NS using a mixed model with random intercept and slope.

**Fig 2:**
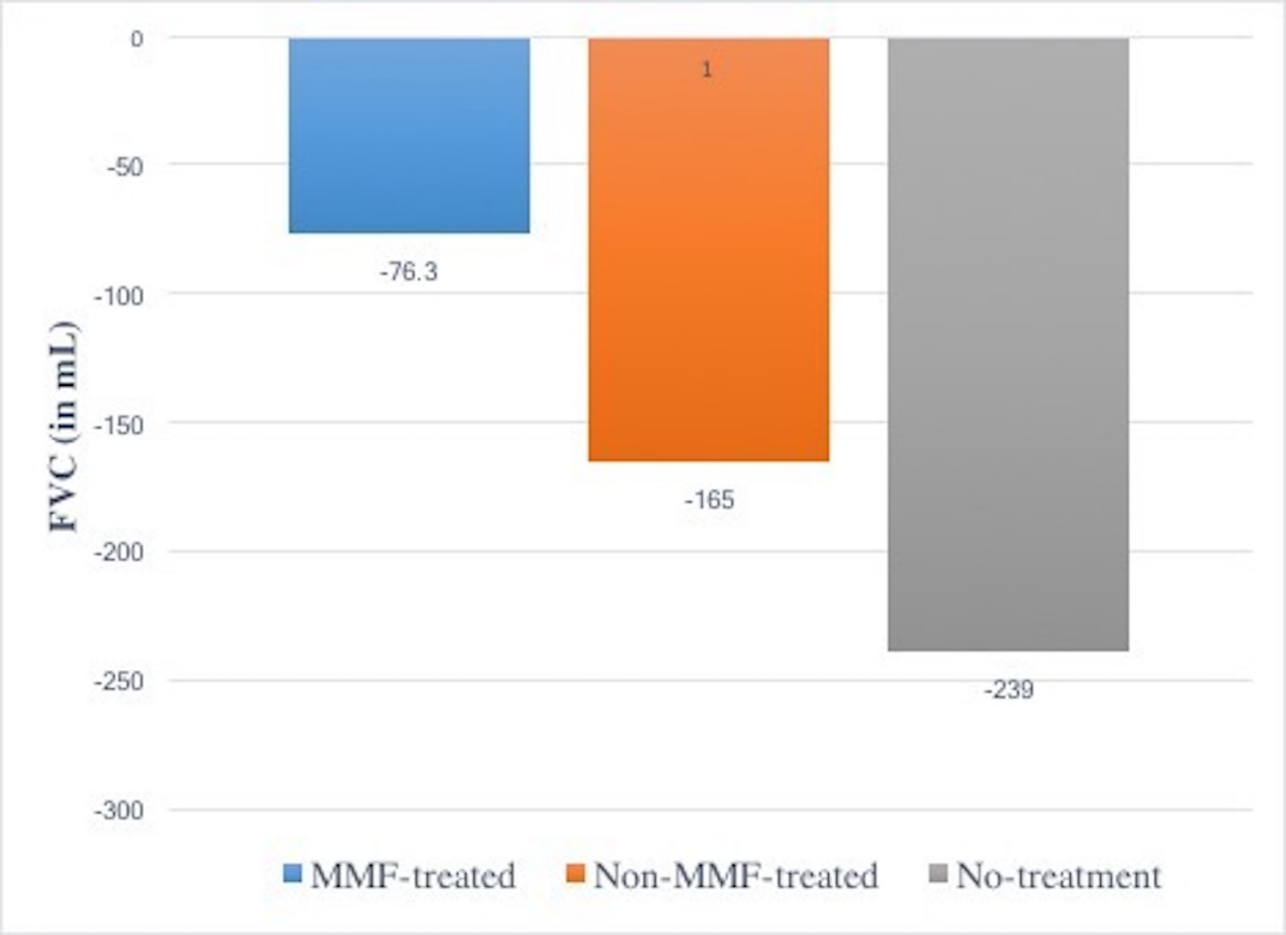
Change in FVC after 12 months between treatment groups. p-value = NS.

The primary effectiveness outcome was the change in FVC at twelve months from baseline. Using a mixed model with random intercept and slope allowing for repeated measures within patients, FVC change after 12 months in the MMF-treated group (-76.3 mL, -2.4% of predicted value) was reduced, but not statistically different, compared to the non-MMF-treated (-165 mL, -8.9% of predicted) and the no-treatment (-239 mL, -11.5% of predicted) groups, respectively (see Figs 1 and 2).

### Categorical change

**Fig 3:**
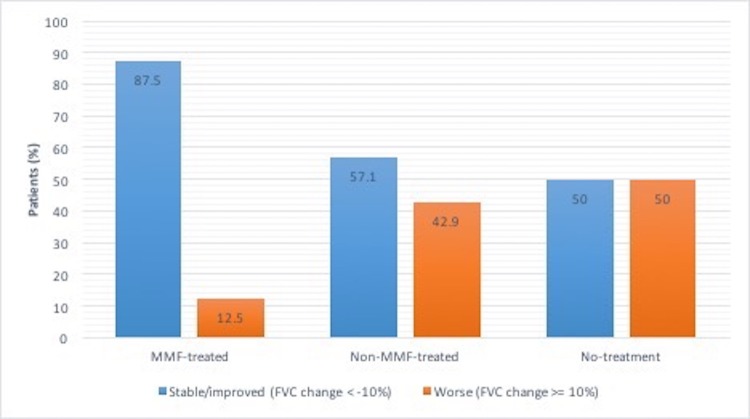
“Stable” versus “worse” FVC at 12 months by treatment group. p-value = NS using Fisher Exact test.

At twelve months, FVC was stable (i.e., absolute decline of less than 10%) in 7 patients (87.5%) in the MMF-treated-group compared to 8 patients (57%) in the non-MMF-treated and 5 patients (50%) in the no-treatment groups, respectively (see Fig 3). Worse FVC (i.e., absolute decline by greater than or equal to 10%) occurred in 1 patient (12.5%) in the MMF-treated group compared to 6 patients (43%) in the non-MMF-treated and 5 patients (50%) in the no-treatment groups, respectively (see Fig 3). Comparisons did not reach statistical significance (P-value of 0.34 using Fisher Exact test).

### Lung transplantation and survival

**Fig 4:**
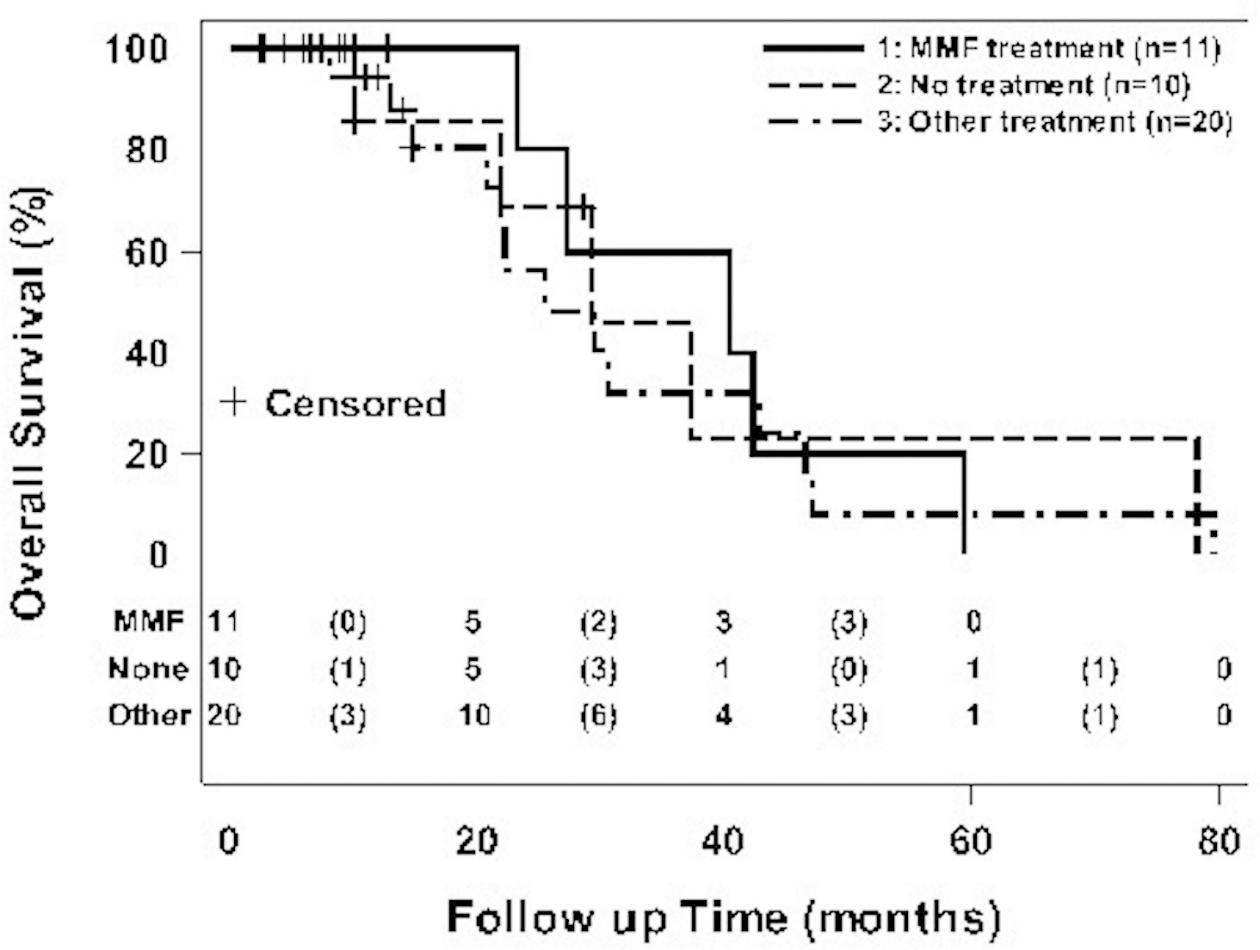
Kaplan-Meier distribution for the probability of overall survival. p-value = NS using log-rank test.

A total of 6 patients underwent lung transplantation: 3 in the MMF-treated, 1 in the non-MMF-treated, and 2 in the no-treatment groups, respectively). Amongst all 41 patients, there were a total of 22 deaths: 4 (36.4%) in the MMF-treated; 13 (65%) in the non-MMF-treated; and 5 (50%) in the no-treatment groups, respectively. Median overall survival was estimated from Kaplan-Meier curves. Median overall survival (censored for lung transplantation) amongst all patients was 29.5 months [95% C.I.: 22 to 42]. The MMF-treated group had a non-significantly greater median overall survival of 40.3 months compared to the non-MMF-treated (25.5 months) and the no-treatment (29.3 months) groups, respectively (see Fig 4).

### DLCO, six-minute walk distance, and GAP scores

Due to significant missing data, diffusion capacity of carbon monoxide (DLCO) and six-minute walk distance could not be analyzed. As a result, gender-age-physiology (GAP) scores also could not be analyzed.

### Adverse events, including AE-IPF

A total of eight acute exacerbations of IPF (AE-IPF) were reported amongst all subjects analyzed: 2 in the MMF-treated, 4 in the non-MMF-treated, and 2 in the no-treatment groups, respectively. There were no statistically significant differences between groups regarding frequency of AE-IPF. In addition, there was only one gastrointestinal adverse event (diarrhea) and no reported episodes of leukopenia, including lymphocytopenia in the MMF-treated group. We did not collect data on kidney function, such as glomerular filtration rates. Overall, very few adverse events were reported likely due to the retrospective design and under-reporting. Therefore, analysis of adverse events in our study is under-powered and any firm conclusions cannot be drawn.

## Discussion

This is the first study of IPF patients treated with MMF in the pre-antifibrotic era that has compared effectiveness and safety to other previously commonly employed treatments or no specific therapy at all. In our study, IPF patients treated with MMF was associated with a potentially clinically important trend towards reduced annual FVC decline, greater FVC stability and improved overall survival. Although we cannot make any direct comparisons between MMF and either antifibrotic pirfenidone or nintedanib, we observed a similar FVC rate of decline with MMF to that reported in the phase three clinical trials of both antifibrotics. As mentioned earlier, in addition to its potent immunosuppressive effects, MMF may also possess important antiproliferative and antifibrotic effects. Future prospective studies with larger sample sizes of IPF patients will be needed to confirm our findings.

Treatment with MMF was generally safe and well tolerated given few reported adverse events; however, there was a low number of total adverse events reported. A recently published systematic review of mycophenolate in systemic sclerosis patients showed that gastrointestinal adverse events were common but not severe enough to preclude its use [43].

Currently available antifibrotic therapies for IPF have been shown to slow down disease progression with an acceptable safety profile; however, both pirfenidone and nintedanib may be extremely costly, especially in the United States, where unsubsidized prices are approximately $7,916 per month and $95,000 per year of treatment. At a U.S. membership warehouse club, MMF may be purchased for approximately $37.24 per 100 500-mg capsules of MMF [44]. At a dose of one gram twice daily (standard dosing), MMF treatment would cost $45 per month and $536 per year. Compared to either pirfenidone or nintedanib, the cost of MMF is lower by approximately $7,900 per month and $94,000 per year for a single patient. This represents a significant potential cost advantage for the use of MMF compared to the recently approved antifibrotic therapies and likely future novel therapeutic agents.

The results of our study should be interpreted in the context of multiple limitations. First, the retrospective design has inherent flaws that are well known such as lack of systematic data acquisition, missing or incomplete data, and under-reporting. As a result, our study’s small sample size had insufficient power to detect statistically significant differences between treatment groups. Second, the baseline FVC of the MMF-treated group was lower, but not statistically different, than the other two groups. Although a lower baseline FVC is usually indicative of more severe disease that would be resistant to medical therapy, the MMF-treated group actually had a trend towards better outcomes. Third, there were more females were in the MMF-treated group than the other two. Although there were no significant differences between groups in the number of patients with positive autoantibodies in the absence of a connective tissue disease, it is possible that these female patients may have had an occult autoimmune disease that may benefit from immunosuppressive therapy. We cannot rule out this possibility without a longer follow-up period and serial autoantibody testing.

Lastly, our results demonstrate that IPF patients treated with MMF may have a potentially clinically important trend towards reduced annual FVC decline, greater FVC stability and improved overall survival compared to proven ineffective/harmful treatment with prednisone, azathioprine, and/or NAC or to no specific treatment. Given MMF’s postulated antiproliferative and antifibrotic mechanisms of action; substantial cumulative clinical experience with its use in various organ transplants, autoimmune diseases, and interstitial lung diseases; a generally favorable side effect profile; significant cost savings compared to presently available antifibrotics; and the potential to stabilize IPF disease progression as demonstrated in our study, we believe that larger, multicenter, prospective studies should be pursued to better evaluate the efficacy and safety of MMF, in combination with currently available antifibrotics, for the treatment of patients with IPF.
